# Multi-Analytic Approach Elucidates Significant Role of Hormonal and Hepatocanalicular Transporter Genetic Variants in Gallstone Disease in North Indian Population

**DOI:** 10.1371/journal.pone.0059173

**Published:** 2013-04-08

**Authors:** Anshika Srivastava, Avshesh Mishra, Rajan Singh, Rajani Rai, Neena Srivastava, Balraj Mittal

**Affiliations:** 1 Department of Genetics, Sanjay Gandhi Post Graduate Institute of Medical Sciences (SGPGIMS) Lucknow, Uttar Pradesh, India; 2 Department of Physiology, King George’s Medical University (KGMU) Lucknow, Uttar Pradesh, India; University of Navarra School of Medicine and Center for Applied Medical Research (CIMA), Spain

## Abstract

**Objective:**

Cholesterol gallstone disease (CGD) is a multifactorial and multistep disease. Apart from female gender and increasing age being the documented non-modifiable risk factor for gallstones the pathobiological mechanisms underlying the phenotypic expression of CGD appear to be rather complex, and one or more variations in genes could play critical roles in the diverse pathways further progressing to cholesterol crystal formation. In the present study we performed genotyping score, Multifactor dimensionality reduction (MDR) and Classification and Regression Tree analysis (CART) to identify combinations of alleles among the hormonal, hepatocanalicular transporter and adipogenesis differentiation pathway genes in modifying the risk for CGD.

**Design:**

The present case-control study recruited total of 450 subjects, including 230 CGD patients and 220 controls. We analyzed common *ESR1, ESR2, PGR, ADRB3, ADRA2A, ABCG8, SLCO1B1, PPARγ2,* and *SREBP2* gene polymorphisms to find out combinations of genetic variants contributing to CGD risk, using multi-analytical approaches (G-score, MDR, and CART).

**Results:**

Single locus analysis by logistic regression showed association of *ESR1* IVS1-397C>T (rs2234693), IVS1-351A>G (rs9340799) *PGR* ins/del (rs1042838) *ADRB3*-190 T>C (rs4994) *ABCG8* D19H (rs11887534), *SLCO1B1* Exon4 C>A (rs11045819) and *SREBP2* 1784G>C (rs2228314) with CGD risk. However, the MDR and CART analysis revealed *ESR1* IVS1-397C>T (rs2234693) *ADRB3*-190 T>C (rs4994) and *ABCG8* D19H (rs11887534) polymorphisms as the best polymorphic signature for discriminating between cases and controls. The overall odds ratio for the applied multi-analytical approaches ranged from 4.33 to 10.05 showing an incremental risk for cholesterol crystal formation. In conclusion, our muti-analytical approach suggests that, *ESR1, ADRB3,* in addition to *ABCG8* genetic variants confer significant risk for cholesterol gallstone disease.

## Introduction

Cholesterol Gallstone disease (CGD) corresponds to one of the most recurrent and costly gastroenterological disorder. It is world-wide health problem representing 10% to 15% of the adult population in industrialised countries [1], [2] whereas a prevalence of 6% have been reported from North India [3]. The female gender and increasing age are the documented non-modifiable risk factors for gallstones [4], the pathobiological mechanisms underlying the phenotypic expression of CGD appear to be rather complex, and one or more defects could occur in genes that play critical roles in the diverse pathways leading to cholesterol gallstone formation. The genetic determinants of gallstone formation have only recently been dissected in humans [5] Compelling evidence for familial clustering and an increased concordance of the trait in monozygotic twins as compared to dizygotic twins [6] further confirms the heritability of gallstones. Thus ‘Gallstone genes’ are continuously being corroborated in genome-wide association studies (GWAS), in case-control cohorts, and in family studies [7], [8].

In human physiology, the gender disparity commences with puberty and continues through the childbearing years [9], [10], [11], [12] which suggests that female sex hormones, could be an important risk factor for the formation of cholesterol gallstones [13]. The actions of these hormones such as estrogen, progesterone and catecholamines are executed through one or more of their respective receptors as estrogen receptors (ESRs), progesterone receptor (PGR) and adrenergic receptor (ADR) Allelic variants of *ESR, PGR* and *ADR* genes have been shown to be associated with susceptibility or progression with various disorders such as myocardial infarction [14], [15], cholesterol gallstones and biliary tract diseases [16].

Another area of interest is hepatocanalicular transporters namely ATP-binding cassette transporters (ABC transporters) and organic anion transporters both encoded by *ABC* and *SLCO1B1* genes respectively. Mutations in genes encoding these transporters have been implicated in cholesterol gallstones formation owing to their ability to influence bile composition and causing retention of substances normally secreted in bile.

Peroxisome proliferator-activated receptor γ 2 (*PPARγ2*) orchestrate the adipocyte differentiation process whereas sterol regulatory element binding protein 2 (*SREBP-2*) is involved in adipocyte differentiation followed by cholesterol homeostasis. Series of previous observations have suggested that regulatory interactions between the *SREBPs* and *PPARγ2* can coordinate cholesterol and fatty acid metabolism. Therefore, sequence variation in these genes may further disrupt the cholesterol homeostasis which in turn may nurture the development of CGD.

Previously, we have studied the role of some individual genetic variants with CGD susceptibility in a North Indian population [17], [18], [19]. Individual SNPs have little predictive value because of their modest effect on risk, but combinations of gene variants may improve the predictive ability and could be used to model susceptibility to CGD. Therefore, the current study aimed to search for gene-gene interactions in the selected pathways (hormonal, hepatocanalicular and adipogenesis differentiation) as a key contributory factor in the disease outcome.

The analysis of such interactions in case-control studies is weighed down by one of the major problems, namely, the curse of dimensionality. Recently, Multifactor-Dimensionality Reduction (MDR) approach, tree-based techniques: classification and regression trees (CART), and genotyping score [20] have been used to detect interactions in large-scale association studies [21]. The strength of these methodologies is their ability to identify association in cases of small sample sizes and low penetrance of candidate single nucleotide polymorphisms (SNPs). Therefore, we have extended our previous work on CGD susceptibility by jointly investigating 13 SNP genotypes in 9 genes belonging to hormonal pathway [*ESR1* IVS1-397C>T (rs2234693), IVS1-351A>G (rs9340799), Ex4-122C>G (rs1801132), *ESR2* -789 A>C (rs1271572), 1082 G>A (rs1256049) *PGR* ins/del (rs1042838) *ADRB3*-190 T>C (rs4994) and *ADRA2A* (rs1800544)], hepatocanalicular transporter pathway [*ABCG8* D19H (rs11887534), *SLCO1B1* Exon4 C>A (rs11045819), Ex6+40T>C (rs4149056)] and adipogenesis differentiation pathway [*PPAR γ2* C>G (rs1801282) *SREBP2* 1784G>C (rs2228314)], avoiding the problem of dimensionality and multiple comparisons.

## Results

### Population Characteristics

The demographic profile of gallstone patients with respect to their age and gender matched controls are presented in Table 1.

**Table 1:** Demographic profile of controls and gallstone patients.

Characteristic	Healthy subjects	Gallstone patients
Total	220	230
Age at interview (years) Mean± SD	49.0±9.8	48.6±11.9
Sex		
Male (n%)	77 (35.0)	83 (36.1)
Female (n%)	143 (65.0)	147 (63.9)

### Allelic Distribution of Studied Polymorphisms in Controls

The genotypic and allelic distribution of *ESR1* IVS1-397C>T, IVS1-351A>G, Ex4-122C>G, *ESR2* -789 A>C, 1082 G>A *PGR* ins/del *ADRB3*-190 T>C and *ADRA2A* -1291 C>G in hormonal pathway, *ABCG8* D19H, *SLCO1B1* Exon4 C>A, Ex6+40T>C in hepatocanalicular transporter pathway and *PPAR γ2* C>G *SREBP2* 1784G>C in adipogenesis differentiation pathway are shown in Table 2, 3 and 4. The details of the selected genes have been shown in supplementary table 1 (Table S1). The observed genotype frequencies of all the studied polymorphisms in controls were in accordance with Hardy-Weinberg equilibrium (p<0.05).

**Table 2:** Hormonal pathway.

Genotypes/Alleles	Controls n (%)	Cases n (%)	p-value	OR (95% CI)
***ESR 1*** ** IVS1-397C>T**				
CC	91 (41.4)	64 (27.8)	−	1 (reference)
CT	110 (50.0)	128 (55.7)	**0.019**	**1.66 (1.09–2.53)**
TT	19 (8.6)	38 (16.5)	**0.001**	**2.98 (1.56–5.70)**
Ptrend			**<0.001**	
*MCS			**0.001**	
CT+TT	129 (58.6)	166 (72.2)	**0.003**	**1.86 (1.23–2.80)**
C	292 (66.4)	256 (55.7)	−	1 (reference)
T	148 (33.6)	204 (44.3)	**0.001**	**1.59 (1.21–2.11)**
***ESR1*** ** IVS1-351A>G**				
AA	90 (40.9)	69 (30.0)		1 (reference)
AG	109 (49.5)	117 (50.9)	0.142	1.37 (0.90–2.07)
GG	21 (9.5)	44 (19.1)	**0.002**	**2.65 (1.43–4.91)**
Ptrend			**<0.001**	
*MCS			**0.001**	
AG+GG	130 (59.1)	161 (70.0)	**0.025**	**1.58 (1.06–2.35)**
A	289 (65.7)	255 (55.5)	−	1 (reference)
G	151 (34.3)	205 (44.5)	**0.005**	**1.49 (1.13–1.95)**
***ESR1*** ** Ex4-122C>G**				
CC	106 (48.2)	120 (52.2)	−	1 (reference)
CG	104 (47.3)	97 (42.2)	0.487	0.87 (0.59–1.29)
GG	10 (4.5)	13 (5.7)	0.981	0.99 (0.41–2.40)
Ptrend			0.605	
*MCS			0.599	
CG+GG	114 (51.8)	110 (47.8)	0.518	0.88 (0.60–1.29)
C	318 (71.9)	337 (73.5)	−	1 (reference)
G	124 (28.1)	123 (26.5)	0.306	0.86 (0.63–1.15)
***ESR2*** ** -789 A>C**				
AA	94 (43.2)	105 (45.7)	−	1 (reference)
AC	109 (49.1)	107 (47.0)	0.596	0.90 (0.61–1.33)
CC	17 (7.7)	18 (7.4)	0.728	0.87 (0.41–1.85)
Ptrend			0.630	
*MCS			0.578	
AC+CC	126 (56.8)	125 (54.3)	0.571	0.90 (0.61–1.31)
A	297 (67.5)	317 (68.9)	−	1 (reference)
C	143 (32.5)	143 (31.1)	0.521	0.91 (0.68–1.21)
***ESR2*** ** 1082 G>A**				
GG	206 (93.6)	212 (92.2)	−	1 (reference)
GA+AA	14 (6.4)	18 (7.8)	0.596	1.22 (0.58–2.56)
Ptrend			0.546	
*MCS			0.435	
G	428 (97.0)	442 (96.3)	−	1 (reference)
A	14 (3.0)	18 (3.7)	0.416	1.36 (0.65–2.87)
***PGR*** ** Ins/Del**				
DD	181 (83.6)	208 (90.4)	−	1 (reference)
DI+II	39 (16.4)	22 (9.6)	**0.009**	**0.46 (0.25–0.82)**
Ptrend			**0.011**	
*MCS			**0.015**	
D	401 (91.1)	438 (95.3)	−	1 (reference)
I	39 (8.9)	22 (4.7)	**0.002**	**0.41 (0.24–0.72)**
***ADRB3 -*** **190 T>C**				
TT	178 (80.9)	158 (68.7)	−	1 (reference)
TC+ CC	42 (19.1)	72 (31.3)	**<0.001**	**1.96 (1.43–2.69)**
Ptrend			**0.003**	
*MCS			**0.002**	
T	398 (90.5)	388 (84.3)	−	1 (reference)
C	42 (9.5)	72 (15.7)	**0.005**	**1.80 (1.19–2.73)**
***ADRA2A*** ** -1291 C>G**				
CC	61 (27.7)	53 (23.0)	−	1 (reference)
CG	117 (53.2)	117 (50.9)	0.678	1.1 (0.7–1.7)
GG	42 (19.1)	60 (26.1)	0.070	1.6 (1.0–2.9)
Ptrend			0.075	
*MCS			0.065	
CG+GG	159 (72.3)	177 (77.0)	0.317	1.2 (0.8–1.9)
C	239 (54.3)	223 (48.4)	−	1 (reference)
G	201 (45.6)	237 (51.5)	0.056	1.5 (1.0–2.5)

MCS = Monte Carlo Simulation; Significant values are in bold; For categorical data Cochrane Armitage trend test was used.

**Table 3:** Hepatocanalicular transporter pathway.

Genotypes/Alleles	Controls n (%)	Cases n (%)	p-value	OR (95% CI)
***ABCG8*** ** 145G>C**				
GG	209 (95.0)	206 (89.6)	−	1 (reference)
GC+CC	11 (5.0)	24 (10.4)	**0.019**	**2.47 (1.16–5.25)**
Ptrend			**0.031**	
*MCS			**0.022**	
G	429 (97.5)	436 (94.9)	−	1 (reference)
C	11 (2.5)	24 (5.1)	**0.025**	**2.41 (1.12–5.22)**
***SLCO1B1*** ** Exon4 C>A**				
CC	205 (93.2)	200 (87.0)	−	1 (reference)
CA+AA	15 (6.8)	30 (13.0)	**0.007**	**2.63 (1.30–5.29)**
Ptrend			**0.028**	
*MCS			**0.020**	
C	425 (96.6)	430 (93.2)	−	1 (reference)
A	15 (3.4)	30 (6.8)	**0.015**	**2.21 (1.16–4.21)**
***SLCO1B1*** ** Ex6+40T>C**				
TT	212 (96.4)	218 (94.8)	−	1 (reference)
TC+CC	8 (3.6)	12 (5.2)	0.422	1.46 (0.57–3.72)
Ptrend			0.416	
*MCS			0.298	
T	432 (99.0)	448 (98.7)	−	1 (reference)
C	8 (1.0)	12 (1.3)	0.850	1.08 (0.46–2.52)

MCS = Monte Carlo Simulation; Significant values are in bold; For categorical data Cochrane Armitage trend test was used.

**Table 4:** Adipogenesis differentiation pathway.

Genotypes/Alleles	Controls n (%)	Cases n (%)	p-value	OR (95% CI)
***SREBP2*** ** 1784G>C**				
GG	145 (65.9)	138 (60.0)	−	1 (reference)
GC	73 (33.2)	82 (35.7)	0.475	1.16 (0.77–1.74)
CC	2 (0.9)	10 (4.3)	**0.045**	**4.87 (1.03–22.96)**
Ptrend			0.067	
*MCS			0.057	
GC+CC	75 (34.1)	92 (40.0)	0.250	1.26 (0.85–1.87)
G	363 (82.5)	358 (77.8)	−	1 (reference)
C	77 (17.5)	102 (22.2)	0.165	1.27 (0.91–1.79)
***PPARG γ2 C>G***				
CC	178 (80.9)	176 (76.5)	−	1 (reference)
CG+GG	42 (19.1)	54 (23.5)	0.351	1.25 (0.78–1.98)
Ptrend			0.256	
*MCS			0.218	
C	398 (90.5)	406 (88.4)	−	1 (reference)
G	42 (9.5)	54 (11.6)	0.652	1.11 (0.71–1.72)

MCS = Monte Carlo Simulation; Significant values are in bold; For categorical data Cochrane Armitage trend test was used.

### Overall Frequency Distribution of Selected Hormonal, Hepatocanalicular Transporter and Adipogenesis Differentiation Gene Polymorphisms in GSD Patients and Healthy Subjects

#### Association of hormonal pathway gene polymorphisms with gallstone patients

Table 2 shows the risk of gallstones in relation to each of the SNPs of *ESR1, ESR2, PGR,* and *ADR* in hormonal pathway. On comparing the genotype frequency distribution of our study groups i.e. gallstone patients with that of healthy subjects (HS), the homozygous variant genotypes of *ESR1* IVS1-397C>T, IVS1-351A>G and *ADRB3* -190 T>C polymorphism showed statistically significant increased risk for developing gallstone (p = <0.001; [OR], 2.9: p = 0.002; [OR], 2.6: p = <0.001; [OR], 1.9.). On the contrary, no significant differences were observed in the distribution of Ex4-122C>G, (ptrend = 0.605; MCS = 0.599), *ESR2* -789 A>C (ptrend = 0.630; MCS = 0.578), Ex6 1082 G>A (ptrend = 0.546; MCS = 0.435), *ADRA2A* -1291 C>G (ptrend = 0.070; MCS = 0.065) polymorphisms in selected groups, both at genotypic and allelic levels. The variant-containing genotypes (DI+II) of *PGR* ins/del showed low risk in gallstone patients which was also significant (p = 0.004; [OR], 0.4; p = 0.009; [OR], 0.4 Table 2) when compared with homozygous wild-type DD genotype. Furthermore, on subdividing the study groups on the basis of gender we observed that *ESR1* IVS1-397C>T and *ADRB3* -190 T>C conferred increased risk for gallstones in female gender (Table S5).

#### Association of hepatocanalicular transporter pathway gene polymorphisms with gallstone patients

Table 3 shows the risk of gallstones in relation to each of the SNPs of *ABCG8* and *SLCO1B1 in* hepatocanalicular transporter pathway. We found that in single locus analysis, the variant genotypes (GC+CC) of *ABCG8* 145 G>C and (CA+AA) of *SLCO1B1* 463 C>A were significantly associated and conferred increased risk of gallstone disease (p = <0.019; [OR], 2.4: p = 0.007; [OR], 2.6). On the contrary, no significant difference were observed in the distribution of *SLCO1B1* 521 T>C (rs4149056) (ptrend = 0.416; MCS = 0.298) polymorphism, both at genotypic and allelic levels and therefore conferred no risk for developing gallstones.

#### Association of adipogenesis differentiation pathway gene polymorphisms with gallstone patients

Table 4 shows the genotype and allele frequency distribution of sequence variants in *SREBP2* 1784 G>C and *PPAR γ2* C>G. A borderline statistical significance was observed when the homozygous variant genotypes of *SREBP2* 1784 G>C (rs2228314) was compared i.e gallstone patients with that of healthy subjects (HS) (p = 0.045; [OR], 4.8). Furthermore, no significant difference was observed in the distribution of *PPAR γ2* C>G (rs1801282) (ptrend = 0.256; MCS = 0.218).

### Haplotype Analysis

#### Linkage disequilibrium and haplotypes analysis of *ESR1* and *ESR2* in case and control groups

On LD analysis, *ESR1* rs2234693 and rs9340799 were found to be in strong linkage disequilibrium (D’ = 0.575). Haplotypes were constructed for the three polymorphisms in *ESR1* gene including IVS1-397C>T, IVS1-351A>G and Ex4-122C>G. The haplotypes comprising the homozygous wild alleles were taken as reference and the difference in the frequencies of haplotypes between patients and controls were tested using chi-square test.

The results of the studied three polymorphisms of *ESR1* revealed that distribution of T,G,C haplotypes was significantly higher in gallstone patients (25.1% v/s 13.7) in comparison to controls and was conferring high risk for gallstone disease (p = 0.0012; [OR], 2.2). Global haplotypes analysis indicated a statistically significant difference between cases and controls based on the distribution pattern of the *ESR1* haplotypes (p = <0.001). Furthermore, none of the *ESR2* haplotypes conferred risk for gallstones presenting the global haplotypes association p-value = 0.65 (Table S2; S3).

#### Linkage disequilibrium and haplotypes analysis of SLCO1B1 in case and control groups

*SLCO1B1* Exon4 C>A and Ex6+40T>C were found to be in strong linkage disequilibrium (D’ = 0.8916). Haplotypes analysis of these two polymorphisms gave rise to three haplotypes, of which C, T was the most common haplotypes in control population. On comparing the haplotypes frequencies in controls and gallstone cases, A, T haplotypes was more commonly distributed in gallstone patients and was imposing risk for the disease (p = 0.017; OR = 2.21) (Table S4).

#### G-score

**Figure 1:**
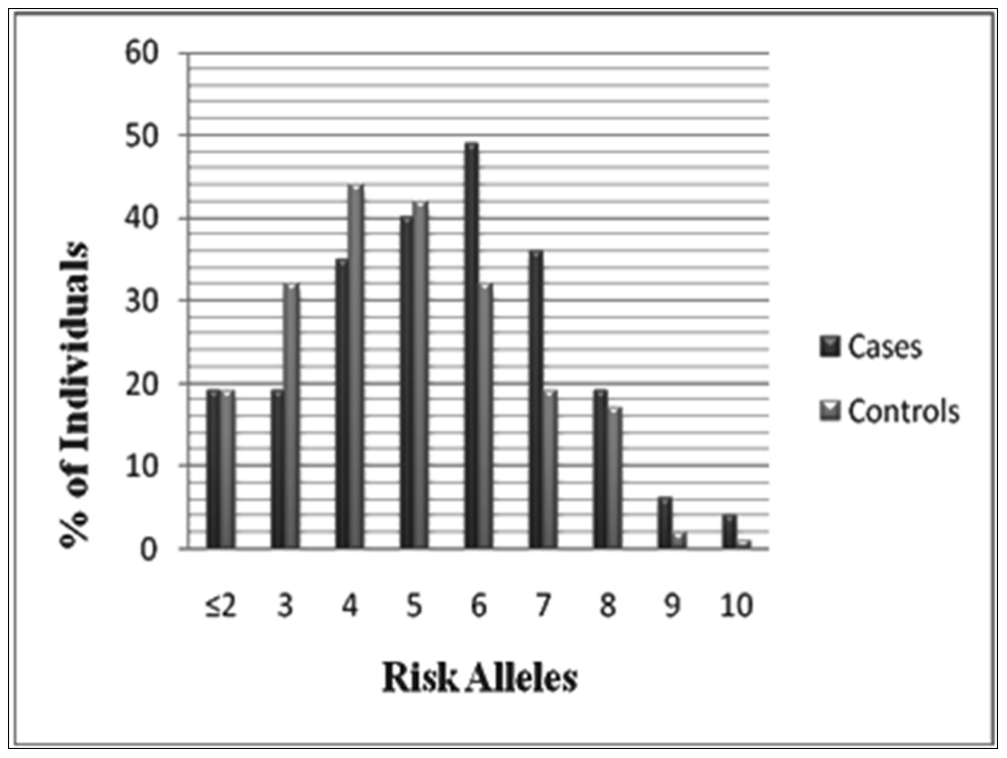
The 13-SNP G-score distribution in patients with gallstones and control subjects.

For each individual, we counted the number of risk-increasing alleles. The number of risk alleles ranged from 1 to 11 in overall 450 subjects (Figure 1). The mean (±SD) G-score was 5.43±1.96 in gallstones subjects and 4.63±1.95 in controls (p-value = <0.001) (Table 5). At the more extreme ends of the risk distribution, CIs around risk estimates became very wide because of small numbers. The number of risk alleles ranged from 1 to 11 with a median of 4 among control subjects and 6 among cases (Figure 1). The risk for gallstone disease was estimated for each number of risk alleles, relative to the median number of risk alleles of 4, and ranged from an OR of 2.27 (95% confidence interval [CI], 1.0–4.6) for 5 risk alleles to an OR of 8.27 (95% CI, 0.90–75.2) for 11 risk alleles. The average relative risk increase per risk allele, when treated as an ordinal variable, however, could be estimated with a high level of precision, and was 2.7 (95% CI, 1.12–1.16). This corresponded to several fold difference in risk between the lowest and the highest number of risk alleles in our population.

**Table 5:** Mean G-Scores in the Selected Pathway and their Corresponding p-values.

Selected Pathways	Cases	Controls	p-value
**Hormonal**	4.46±1.68	3.92±1.76	**0.001**
**Hepatocanalicular Transporter**	0.29±0.54	0.168±0.37	**0.004**
**Adipogenesis Differentiation**	0.67±0.84	0.54±0.70	0.062
**Overall Genotyping Score Mean for all pathways**	5.43±1.96	4.63±1.95	**<0.001**

Significant values are in bold.

### Association of High-Order Interactions with GSD Risk by MDR Analysis

Table 6 shows the best interaction model by MDR analysis. The one-factor model for predicting GS risk was *ABCG8* 145 G>C SNP (testing accuracy = 0.515, CVC = 7/10, permutation p = 0.027). The two-factor model of *ESR1* IVS1 351A>G and *ADRB3 -*190 T>C had an improved testing accuracy of 0.578 (permutation p = <0.001) however, the CVC was increased (10/10). The three factor model was the three-factor model including *ESR1* IVS1-397C>T *ESR1* IVS1 351A>G and *ADRB3* -190T>C SNPs, which yielded the testing accuracy of 0.605 and the CVC of 08/10 (permutation p = <0.001). The best four-factor interaction model consisted of *ESR1* IVS1-397C>T, *ESR1* IVS1 351A>G, *ADRB3 -*190T>C and *ABCG8* 145 G>C with highest testing accuracy compared with the one-factor model (CVC = 10/10 permutation p = <0.001).

**Table 6:** Association of High-Order Interactions with GSD Risk by MDR Analysis.

No. of interactingloci	Best Interaction Model	Testing Accuracy	#CVC	*P* for permutation Testing
1	*ABCG8* 145 G>C	0.5156	7/10	**0.0027**
2	*ESR1* IVS1 351A>G *ADRB3* -190T>C	0.5784	10/10	**<0.001**
3	*ESR1* IVS1-397C>T *ESR1* IVS1 351A>G, *ADRB3* -190T>C	0.6050	8/10	**<0.001**
4	a *ESR1* IVS1-397C>T, *ESR1* IVS1 351A>G, *ADRB3 -*190T>C*ABCG8* 145 G>C	0.6212	10/10	**<0.001**

# CVC: Cross Validation Consistency.

a The model with the maximum testing accuracy and maximum CVC cross was considered as the best model. The present study calculated, the best interaction model as the four-factor model including *^a^ESR1* IVS1-397C>T, *ESR1* IVS1 351A>G, *ADRB3*-190T>C, *ABCG8* 145 G>C polymorphisms.

### Association of High-Order Interactions with GSD Risk by CART Analysis

Table 7 shows the CART, which included all investigated genetic variants of the selected pathways. The final tree structure contained seven terminal nodes as defined by single-nucleotide polymorphisms of the hormonal, hepatocanalicular transporter and adipogenesis differentiation pathway genes. Consistent with the MDR best one-factor model, the initial split of the root node on the decision tree was *ESR1* IVS1-397C>T, suggesting that this SNP is the strongest risk factor for GSD among the polymorphisms examined. Individuals carrying *ESR1* IVS1 -397CC, *ADRB3*-190 TT, *ABCG8* 145 GG and *ADRA2A* GG genotypes had the lowest case rate of 17.2%, considered as reference. Further inspection of the tree structure revealed distinct interaction patterns between individuals carrying the *ESR1* IVS1-397 variant and those with the *ADRB3* variant and *SLCO1B1* 463 C>A wild genotypes. Using the terminal node with lowest case rate as reference, individuals carrying the combination of *ESR1* IVS1-397TT, *SLCO1B1* Exon4CC, *ESR2* 1082GG, *ESR1* IVS1-351AA and *ESR1* Ex4-122GG exhibited a significantly higher risk for GSD (adjusted OR 5.083; 95% CI, 1.3–18.48), whereas individuals with the combined genotypes of *ESR1* IVS1-397TT, *ABCG8* 145 GC+CC, *ESR2* 1082GG*+ ESR1* IVS1-351GG and *ADRB3* TC+CC had the highest risk for CGD (adjusted OR 6.48; 95% CI, 1.9–22.08). (Table 7).

**Table 7:** Risk Estimates of CART Terminal Nodes.

Nodes	Genotypes	Case rate^a^ (%)	p-value	OR^b^
1	*ESR1IVS1397W+ADRB3W+ABCG8W+ADRA2A W*	17.2	−	**Reference**
2	*ESR1IVS1-397V+SLCO1B1 463 W+ESR2 1082W+ ESR1IVS1-351V +ESRHinf1 W*	21.7	0.352	0.429 (0.07–2.55)
2	*ESR1IVS1-397W+ ADRB3W+ABCG8W +ADRA2AV+ESR2Bsa1 V*	29.3	0.475	1.576 (.453–5.479)
3	*ESR1IVS1-397W+ ADRB3W+ABCG8W +ADRA2AV+ESR2Bsa1W*	47.2	**0.018**	**4.33 (1.29–14.59)**
4	*ESR1IVS1-397V+SLCO1B1463 W+ESR21082W+ESR1IVS1-351V +ESRHinf1 V*	56.2	**0.014**	**5.083 (1.39–18.48)**
5	*ESR1IVS1397V+ABCG8V+ESR21082W+ESR1IVS1-351V+ADRB3V*	63.0	**0.003**	**6.48 (1.90–22.08)**
6	*ESR1IVS1-397V+ ADRB3V*	76.9	**0.002**	**10.05 (2.33–43.29)**
7	*ESR1IVS1-397W+ADRB3V+ ESRHinf1W*	78.6	**0.002**	**24.554 (3.24–185.84)**

W = wild genotype. V = variant genotype.

a Case rate is the percentage of gallstone patients among all individuals in each node.

b ORs of terminal nodes were calculated by LR analysis adjusted for age and gender.

Significant values are in bold.

## Discussion

In order to achieve a more comprehensive evaluation of CGD risk, present analysis was performed in order to identify high and low intrinsic risk sets of sequence variants. Of the included 13 polymorphisms, some of them were found to be significantly associated with CGD risk in our previous studies [17], [18], [19] while others showed little or no influence on the risk for CGD development. Moreover, accumulating evidence supports the importance of adipogenesis differentiation and adrenergic receptor pathways in cholesterol associated diseases [22], [23], [24]. Therefore, we further extended our work by incorporating these two pathways.

In the single-locus analysis, genetic variants of hormonal pathway, *ESR1* IVS1-397C>T, IVS1-351A>G and *ADRB3* 190 T>C were significantly associated with GSD risk [17]. However, *Alu* insertion polymorphism of progesterone receptors (*PGR*) conferred lower risk with gallstones. In hepatocanalicular transporter pathway *ABCG8* D19H and *SLCO1B1* Exon4 C>A conferred increased risk for CGD At haplotypes level, we found that the gallstones subjects who carry *ESR1* haplotypes IVS1-397T, IVS1-351G, Ex4-122C and *SLCO1B1* haplotypes Exon4A, Ex6+40T conferred increased risk for gallstones.

Based on the candidate SNPs in genes involved in the gallstone pathway. We created a consolidated Genotype Score (G-score) from the number of risk alleles as previously reported for risk assessment of cardiovascular events and diabetes [25], [26]. Our assumption was that individuals with a high G-score might have a higher probability of gallstone development as compared to those with the low G-score. The overall G-scores for the three selected pathways obtained were highly significant and conferred increased risk for gallstone development. Further calculating the G-score individually in respective pathways we found both hormonal and hepatocanalicular transporter pathway conferred increase risk. These results suggest significant role of hormonal receptor and hepatocanalicular transporters in gallstone disease.

For the higher order gene-gene interaction analysis, we employed statistical approaches namely MDR and CART analysis to find out the particular combinations of genetic variants contributing to CGD risk. In MDR analysis, we observed the best four-factor interaction model consisting of *ESR1* IVS1-397C>T, *ESR1* IVS1 351A>G, *ADRB3 -*190T>C and *ABCG8* 145 G>C with highest testing accuracy compared with the one-factor model.

In CART analysis, which is a non-parametric statistical approach for conducting regression and classification analyses by recursive partitioning. [17], study subjects were grouped according to different risk levels on the basis of the different gene polymorphisms. From this analysis, we found that development of CGD involves complex genetic interactions among the hormonal and hepatocanalicular transporter genetic variants. As our results from CART analyses consistently suggested that *ESR1*IVS1-397TT, *ABCG8*GC+CC, *ESR1*IVS1-351GG and *ADRB3* TC+CC polymorphisms are the most important single susceptibility factor for CGD development.

The association between hormonal receptor gene polymorphisms and risk of gallstones are biologically convincing. It has been assumed that the gallbladder is a female sex hormone responsive organ, and these hormones might be involved in the pathogenesis of gallbladder diseases. Elaborating on estrogen receptor the animal studies have shown that ESRs are present in the hepato-pancreatic-biliary tree [27], [28], [29] including bile duct epithelial cells and gallbladder. In addition, immunohistochemical and quantitative RT PCR studies have also revealed that the expression level of *ESR1* gene is approximately 50 fold higher compared to *ESR2*. In animal models, 17beta estradiol promoted gallstone formation which further involves the upregulation of hepatic expression of ERalpha but not ERbeta. These studies show that *ESR-1* is key player and findings may offer a new approach to treat gallstones by inhibiting hepatic ER activity with a liver-specific, ERalpha-selective antagonists.

The literature regarding the *ADRB3* confirms that it is localized in the smooth muscles of the vasculature and the muscularis propria of the gallbladder [30] where it is thought to mediate relaxation and increase mucosal blood flow. The T>C polymorphism results in lowered responsiveness to potent agonists including endogenous catecholamines. [31] The mutated receptor had less ability to stimulate adenylyl cyclase and therefore less accumulation of cAMP. [31] Activation of *ADRB3* also results in smooth muscle relaxation in the guinea-pig common bile duct, [32] and since the ductal smooth muscle appear to be more sensitive to activation of the ß3-adrenoceptor, there is the possibility that these receptors may be involved in the regulation of tone in the ductal smooth muscle and hence the outflow of bile. Thus the inhibiting variant C in *ADRB3* might result in gallstone formation by impairing the relaxation of the gallbladder and probably the biliary tree too, setting the stage for crystal formation.

In the selected hepatocanalicular transporters *ABCG8* 145G>C conferred increased risk both individually and in combination to hormonal receptors. A genome wide scan carried out by Buch *et al.*, [33] identified a variant D19H in the hepatic cholesterol transporter (*ABCG8*) as major susceptibility factor for human gallstone disease. Subsequently, this association has been replicated in various populations [9], [19], [34], [35].

The phenomenon that a combination of polymorphisms within genes of unrelated pathways may elevate the risk for CGD could be explained by two hypotheses. One possibility is that some connection between these genes or proteins exists but still remains to be discovered. Another hypothesis, more credible in our opinion, is that the genes influencing risk for CGD may as well comprise a set of alterations located within genes not related to each other.

Our multi-analytic approach revealed that the combination of genotypes of respective polymorphisms as *ESR1* IVS1-397 variant, *ABCG8* 145 variant, *ESR1* IVS1-351 variant and *ADRB3* 190 variant pose a significant risk for developing gallstone. Comparing to the results of single locus analysis the role of *SLCO1B1* Exon4 C>A *SREBP2* 1784 G>C and *PGR* ins/del was diminished when the overall analysis of 13 selected polymorphisms was performed. It also suggests that *ESR1, ADRB3* and *ABCG8* have significant incremental risk factors for gallstone disease. Thus, the application of these multi analytical approaches allowed creating a decision that has more sensitivity or specificity and was more accurate with reasonable power as compared to single strategy employed in calculating risk allele for disease prediction.

Also a prominent significant role of hormonal pathway was elucidated when the means of genotyping scores of selected pathways was calculated separately or all together. Therefore, exhaustive analysis of multi-analytic approaches as MDR, CART and G-scores are well recognized methods in understanding complex traits, such as disease susceptibility and also the etiology of complex diseases.

In summary, this is the first comprehensive study to use a multigenic analysis for cholesterol gallstone disease, and the data suggest that individuals with a higher number of genetic variations in hormonal and hepatocanalicular transporter pathway genes are at an increased risk for cholesterol gallstone disease, confirming the importance of taking a multigenic pathway based approach to risk assessment. The finding also indicates that the development of gallstone involves complex genetic interactions and follows different pathways depending on the specific genetic background of the subjects. The present study provided evidence supporting the cholesterol supersaturation contribution of hormonal, hepatocanalicular transporter and adipogenesis differentiation pathway genes, of which interaction between *ESR1, ADRB3* and *ABCG8* genes were the most important.

Thus, our results support the concept that genetic polymorphisms can be used as cholesterol gallstone risk predictors and multiple polymorphisms allow more precise delineation of risk groups and suggest the future direction of association studies. However, the present study included only North Indian individuals, therefore the results need to be replicated in other ethnic groups.

## Patients and Methods

### Ethics Statement

The institutional ethical committee of Sanjay Gandhi Post Graduate Institute of Medical Sciences (SGPGIMS) approved the present study protocol and the authors followed the norms of World’s Association Declaration of Helsinki. All the participants provided written informed consent.

### Study Population

The case control study recruited a total of 450 subjects, including 230 cholesterol gallstone patients (GS) and 220 healthy subjects. From the year June 2006 to September 2011 symptomatic cholesterol GS patients attending the Department of Gastro-surgery, Sanjay Gandhi Post Graduate Institute of Medical Sciences and Department of Surgical Oncology Lucknow India, were approached for participation in the present study. All subjects were unrelated and confirmed to North Indian ethnicity.

#### Phenotype data

For each individual, ultrasound examinations were conducted at the Department of Radio-diagnosis and Imaging SGPGIMS, Lucknow. Participants were considered as having gallstones when one of the subsequent diagnostic criteria was satisfied: (1) Gallbladder lumen with mobile nodular or dependent layering echoes that exhibited posterior acoustic shadowing, or (2) Gallbladder with hyperechoic shadowing material filling the gallbladder lumen with an appearance of the WES triad (i.e., the gallbladder wall, the echo of the stone, and the acoustic shadow–a specific ultrasonographic sign of gallstones used to make a reliable diagnosis of cholelithiasis [36]. The healthy controls were randomly selected from a pool of healthy volunteers that visited the general health check-up center at SGPGIMS Lucknow, during the same period. In addition to a self-reported gallstone history, transabdominal ultrasound was performed to validate gallstone status and to identify silent gallstones. Inclusion criteria for controls also included absence of asthma, coronary artery disease, diabetes mellitus determined through maternal and paternal family history. At recruitment, informed consent was obtained from each subject and the information on demographic characteristics, such as sex and age was collected by questionnaire. Both patients and controls had similar ethnicity. The blood sample and the clinical details were collected from each participant at recruitment.

### DNA Samples and Genotyping

Genomic DNA was isolated from peripheral blood leukocytes using salting out method [37]. The polymorphisms were genotyped using the PCR or PCR restriction fragment length polymorphism method. The details of genotyping for studied polymorphisms are shown in Table S1. Ten percent of masked, random sample of cases and controls were tested twice by different laboratory personnel and the reproducibility was 100%.

### Genotype Score Calculation (G-score)

A Genotype score (G-score) was defined as the cumulative number that counts the total number of risk-increasing alleles in individuals. Genotyping of 13 selected SNPs in candidate genes involved in hormonal, hepatocanalicular and adipogenesis differentiation pathways was performed and G-score was computed from the number of variant alleles. A value of 2, 1 and 0 was allotted to homozygous variant, heterozygous and homozygous wild type genotypes respectively. Variant genotype was considered as risk conferring. Using these 13 SNPs a Genotype Score (G-score) was constructed ranging from 0 to 26 on the basis of the number of risk alleles. For each sample a consolidated G-score was calculated by adding the values from all 13 SNPs together.

### Statistical Analysis

Descriptive statistics were presented as mean and standard deviation [SD] for continuous measures while absolute value and percentages were used for categorical measures. Differences in genotype and allele frequencies between study groups were estimated by chi-square test. Unconditional logistic regression was used to estimate odds ratios [ORs] and their 95% confidence intervals [CIs] adjusting for age and sex. A two-tailed p-value of less than 0.05 was considered a statistical significant result. All statistical analyses were performed using SPSS software version 16.0 (SPSS, Chicago, IL, USA). Ptrend and Monte Carlo Simulation (MCS) were calculated through Cochrane Armitage trend using XLstats whereas haplotype analysis was performed using SNPstats (http://bioinfo.iconcologia.net/SNPstats).

Furthermore, higher-order gene-gene interactions associated with CGD risk were determined through multifactor dimensionality reduction (MDR) using software version 2.0 beta8 and classification regression tree analysis (CRT) using SPSS software version 16.0.

## Supporting Information

Table S1 **The genes and SNPs investigated.**
(DOC)

Table S2 **Haplotypes association of** ***ESR1*** **gene (age and gender adjusted).**
(DOC)

Table S3 **Haplotypes analysis of** ***ESR2*** **gene (age and gender adjusted).**
(DOC)

Table S4 **Haplotypes analysis of** ***SLCO1B1*** **gene (age and gender adjusted).**
(DOC)

Table S5 **Odds Ratios and 95% CI for Gallstones in Relation to Polymorphisms of Hormonal Pathway after Subdividing on the Basis of Gender.**
(DOC)
