# Supplementary material for: Multi-Analytic Approach Elucidates Significant Role of Hormonal and Hepatocanalicular Transporter Genetic Variants in Gallstone Disease in North Indian Population
Source: PLoS One. 2013 Apr 8;8(4):e59173. doi: 10.1371/journal.pone.0059173 (PMC3620121; doi:10.1371/journal.pone.0059173)
Supplement: Table S1 — The genes and SNPs investigated. (DOC) [file pone.0059173.s001.doc]

**Table S1.** The genes and SNPs investigated

| **Gene** | **Polymorphism** | | **Forward primer (5′-3′)** | **Reverse primer (5′-3′)** | **AP^*^** | **RE^#^** | **Fragments** |
| --- | --- | --- | --- | --- | --- | --- | --- |
| *ADRB3* | | rs4994 T>C | CCAGTGGGCTGCCAGGGG | GCCAGTGGCGCCCAACGG | 158 | *BstN1* | T =97+64+61bp  C=158bp |
| *ADRA2A* | | rs1800544 C>G | GGAGGTTACTTCCCTCG | GGTACCTTGAGCTAGAGAC | 209 | *Msp1* | C = 34+175 bp  G = 34+55+120 bp |
| *ESR1* | | rs2234693 C>T | ATCCAGGGTTATGTGGCAATGACG | TCAGATAATCGACGCCAGGGT | 526 | *PvuII* | C = 426 + 100bp  T = 526bp |
| *ESR1* | | rs9340799 A>G | ATCCAGGGTTATGTGGCAATGACG | TCAGATAATCGACGCCAGGG | 526 | *Xba1* | A = 526bp  G = 379+147bp |
| *ESR1* | | rs1801132 C>G | AGCCCGCTCATGATCAAACG | GGATCATACTCGGAATAGAGAAT | 120 | *Hinf1* | C =120bp  G = 99+21bp |
| *ESR2* | | rs1271572 A>C | CTGCCCACCCCTCTTCTC | CCATCTTTGGAGCCTGTCTT | 454 | *Bsa1* | A = 454bp  C= 256 + 198bp |
| *ESR2* | | rs1256049 G>A | CAGGCTTTGTGGAGCTCAG | ACCTGTCCAGAACAAGATCT | 156 | *Rsa1* | G = 156bp  A = 125+31bp |
| *PR* | | rs1042838 306-bp insertion | GCCTCTAAAATGAAAGGCAGAAAGC | GCGCGTATTTTCTTGCTAAATGTCTG | 175 | *-* | D = 175bp  I = 481bp |
| *SLCO1B1* | | rs11045819 C>A | ATAATGGTGCAAATAAAGGGG | ACCTTTTCCCACTATCTCCG | 224 | *Msp1* | C = 205+ 19bp  A = 224bp |
| *SLCO1B1* | | rs4149056 T>C | TTGTCAAAGTTTGCAAAGTG | GAAGCATATTACCCATGAGC | 209 | *Hha1* | T = 209bp  C=189 bp and 20 |
| *ABCG8* | | rs11887534 G>C | ATGGCCGGGAAGGCGGCAGAGGAGAG | ACTTCCCATTGCTCACTCACCGAGGGAT | 83 | *BamHI* | G = 56+27bp  C = 83bp |
| *SREBP2* | | rs2228314 G>C | GCCAGTGACCATTAACACCTTTTGA | TCGTCTTCAAAGCCTGCCTCAGTGGCTGGC | 247 | *Msp1* | G = 85+162bp  C = 247bp |
| *PPAR-γ2* | | rs1801282 C>G | GCCAATTCAAGCCCAGTC | GATATGTTTGCAGACAGTGTATCAGTGAAGGAATCGCTTTCC | 273 | *BstUI* | C = 273bp  G = 227+46bp |

***Amplified Product #Restriction Enzyme**
