# Supplementary material for: Multi-Analytic Approach Elucidates Significant Role of Hormonal and Hepatocanalicular Transporter Genetic Variants in Gallstone Disease in North Indian Population
Source: PLoS One. 2013 Apr 8;8(4):e59173. doi: 10.1371/journal.pone.0059173 (PMC3620121; doi:10.1371/journal.pone.0059173)
Supplement: Table S2 — Haplotypes association of ESR1 gene (age and gender adjusted). (DOC) [file pone.0059173.s002.doc]

**Table S2. Haplotype association of *ESR1* gene (age and gender** adjusted)

| **Gallstone patients and Controls** | | | | | |
| --- | --- | --- | --- | --- | --- |
|  | **GS (%)** | | **HC (%)** | p-value | Odds Ratio OR (95% CI) |
| C_rs2234693_ A_rs9340799_ C rs_1801132_ | | 0.334 | 0.3783 | --- | 1 (reference) |
| T_rs2234693_ G_rs9340799_ C_rs1801132_ | | 0.251 | 0.1371 | **0.0012** | **2.22 (1.37 - 3.59)** |
| C_rs2234693_ A_rs9340799_ G_rs1801132_ | | 0.1034 | 0.1437 | 0.61 | 0.84 (0.44 - 1.62) |
| C_rs2234693_ G_rs9340799_ C_rs1801132_ | | 0.0831 | 0.101 | 0.7 | 1.13 (0.62 - 2.04) |
| T_rs2234693_ A_rs9340799_ C_rs1801132_ | | 0.0645 | 0.1018 | 0.52 | 0.78 (0.37 - 1.66) |
| T_rs2234693_ G_rs9340799_ G_rs1801132_ | | 0.0755 | 0.0643 | 0.2 | 1.56 (0.79 - 3.07) |
| C_rs2234693_ G_rs9340799_ G_rs1801132_ | | 0.0524 | 0.0331 | 0.18 | 1.91 (0.75 - 4.86) |
| T_rs2234693_ A_rs9340799_ G_rs1801132_ | | 0.036 | 0.0407 | 0.89 | 0.94 (0.38 - 2.34) |
| **Global haplotype association p-value: 0.0042** | | | | | |

Significant values are in bold
