# Supplementary material for: Multi-Analytic Approach Elucidates Significant Role of Hormonal and Hepatocanalicular Transporter Genetic Variants in Gallstone Disease in North Indian Population
Source: PLoS One. 2013 Apr 8;8(4):e59173. doi: 10.1371/journal.pone.0059173 (PMC3620121; doi:10.1371/journal.pone.0059173)
Supplement: Table S3 — Haplotypes analysis of ESR2 gene (age and gender adjusted). (DOC) [file pone.0059173.s003.doc]

**Table S3. Haplotype analysis of *ESR2* gene (age and gender adjusted)**

| **Haplotypes** | **GS (%)** | **HC (%)** | p-value | Odds Ratio OR (95% CI) |
| --- | --- | --- | --- | --- |
| G_rs1256049_ A_rs1271572_ | 0.6595 | 0.6611 | --- | 1(reference) |
| G_rs1256049_ C _rs1271572_ | 0.3014 | 0.3071 | 0.91 | 0.98 (0.72 - 1.34) |
| A_rs1256049_ A_rs1271572_ | 0.0274 | 0.0139 | 0.25 | 1.99 (0.61 - 6.46) |
| A _rs1256049_ C _rs1271572_ | 0.0117 | 0.0179 | 0.55 | 0.66 (0.17 - 2.57) |
| Global haplotype association p-value: 0.65 | | | | |
