# Supplementary material for: Multi-Analytic Approach Elucidates Significant Role of Hormonal and Hepatocanalicular Transporter Genetic Variants in Gallstone Disease in North Indian Population
Source: PLoS One. 2013 Apr 8;8(4):e59173. doi: 10.1371/journal.pone.0059173 (PMC3620121; doi:10.1371/journal.pone.0059173)
Supplement: Table S4 — Haplotypes analysis of SLCO1B1 gene (age and gender adjusted). (DOC) [file pone.0059173.s004.doc]

**Table S4. Haplotype analysis of *SLCO1B1* gene (age and gender** adjusted)

| **Haplotypes** | **GS (%)** | **HC (%)** | p-value | Odds Ratio OR (95% CI) |
| --- | --- | --- | --- | --- |
| C _rs11045819_ T_rs4149056_ | 0.9046 | 9409 | --- | 1 (reference) |
| A_rs11045819_ T_rs4149056_ | 0.0694 | 0.0341 | **0.017** | **2.21 (1.16 - 4.24)** |
| C_rs11045819_ C _rs4149056_ | 0.0259 | 0.025 | 0.77 | 1.14 (0.48 - 2.70) |
| Global haplotype association p-value: 0.043 | | | | |

Significant values are in bold
