# Supplementary material for: Multi-Analytic Approach Elucidates Significant Role of Hormonal and Hepatocanalicular Transporter Genetic Variants in Gallstone Disease in North Indian Population
Source: PLoS One. 2013 Apr 8;8(4):e59173. doi: 10.1371/journal.pone.0059173 (PMC3620121; doi:10.1371/journal.pone.0059173)
Supplement: Table S5 — Odds Ratios and 95% CI for Gallstones in Relation to Polymorphisms of Hormonal Pathway after Subdividing on the Basis of Gender. (DOC) [file pone.0059173.s005.doc]

**Table S5. Odds Ratios and 95% CI for Gallstones in Relation to Polymorphisms of Hormonal Pathway after Subdividing on the Basis of Gender**

| **Polymorphism** | **Controls n (%)** | **GS^$^ n (%)** | **OR^*^** | | **CI^#^** | | | | **p-value** |
| --- | --- | --- | --- | --- | --- | --- | --- | --- | --- |
| ***ESR1* IVS1-397C>T** | | | | | | | | | |
| **Male** |  |  |  | | |  | | |  |
| CC | 39 (50.6) | 35 (42.2) | 1 (reference) | | | - | | | - |
| CT | 33 (42.9) | 40 (48.2) | 1.5 | | | 0.6–3.6 | | | 0.32 |
| TT | 5 (6.5) | 8 (9.6) | 3.1 | | | 0.7-13.8 | | | 0.13 |
| C | 111 (72.7) | 110 (66.3) | 1 (reference) | | | - | | | - |
| T | 43 (27.3) | 56 (33.7) | 1.4 | | | 1.0–1.9 | | | 0.05 |
| **Female** | | | | | | | | | |
| CC | 52 (36.4) | 35 (23.8) | 1 (reference) | | | | | - | - |
| CT | 77 (53.8) | 88 (59.9) | 1.6 | | | | | 0.9-2.7 | 0.08 |
| TT | 14 (9.8) | 24 (16.3) | **2.3** | | | | | **1.0–5.0** | **0.04** |
| C | 181 (63.3) | 158 (54.1) | 1 (reference) | | | | |  |  |
| T | 105 (36.7) | 136 (45.9) | **2.1** | | | | | **1.3–3.4** | **0.02** |
| ***ESR1* IVS1-351A>G** | | | | | | | | | |
| **Male** |  |  |  | | | |  | |  |
| AA | 33 (42.9) | 38 (45.7) | 1 (reference) | | | | - | | - |
| AG | 39 (50.6) | 41 (50.0) | 2.0 | | | | 0.8–5.1 | | 0.11 |
| GG | 5 (6.5) | 4 (4.3) | 1.0 | | | | 0.6-1.4 | | 0.98 |
| A | 105 (68.1) | 117 (70.4) | 1 (reference) | | | |  | |  |
| G | 49 (31.8) | 49 (29.5) | 1.5 | | | | 0.5–3.1 | | 0.87 |
| **Female** |  |  |  | | | |  | |  |
| AA | 57 (39.9) | 51 (34.7) | 1 (reference) | | | | - | | - |
| AG | 70 (49.0) | 76 (51.7) | 1.2 | | | | 0.7–2.0 | | 0.50 |
| GG | 16 (11.2) | 20 (13.6) | 1.3 | | | | 0.6-2.8 | | 0.47 |
| A | 184 (64.3) | 178 (59.5) | 1 (reference) | | | | - | | - |
| G | 102 (35.7) | 116 (40.5) | 1.8 | | | | 1.9–5.3 | | 0.38 |
| ***ESR1* Ex4-122C>G** | | | | | | | | | |
| **Male** |  |  |  | | | |  | |  |
| CC | 45 (58.4) | 47 (56.6) | 1 (reference) | | | | - | | - |
| CG | 32 (41.6) | 30 (36.1) | 1.0 | | | | 0.4–2.3 | | 0.87 |
| GG | 0 | 6 (7.2) | - | | | | - | | - |
| C | 122 (79.2) | 124 (74.6) | 1 (reference) | | | | - | | - |
| G | 32 (20.7) | 42 (25.3) | 1.6 | | | | 0.9–2.6 | | 0.12 |
| **Female** |  |  |  | | | |  | |  |
| CC | 61 (42.7) | 73 (49.7) | 1 (reference) | | | | - | | – |
| CG | 72 (50.3) | 67 (45.6) | 0.9 | | | | 0.5–1.4 | | 0.47 |
| GG | 10 (7.0) | 7 (4.8) | 0.5 | | | | 0.1-1.4 | | 0.16 |
| C | 194 (67.8) | 213 (72.4) | 1 (reference) | | | |  | | - |
| G | 92 (32.1) | 81 (27.5) | 2.3 | | | | 1.2–4.2 | | 0.26 |
| ***ESR2* -789 A>C** | | | | | | | | | |
| **Male** |  |  |  | | | |  | |  |
| AA | 33 (42.9) | 36 (43.4) | 1 (reference) | | | | - | | – |
| AC | 39 (50.6) | 42 (50.6) | 0.8 | | | | 0.3–2.0 | | 0.78 |
| CC | 5 (6.5) | 5 (6.0) | 0.7 | | | | 0.1-3.9 | | 0.74 |
| A | 105 (68.1) | 114 (68.6) | 1 (reference) | | | |  | | - |
| C | 49 (31.8) | 52 (31.3) | 1.0 | | | | 0.7–1.7 | | 0.82 |
| **Female** |  |  |  | | | |  | |  |
| AA | 61 (42.7) | 68 (46.3) | 1 (reference) | | | | - | | - |
| AC | 70 (49.0) | 66 (44.9) | 1.0 | | | | 0.6–1.6 | | 0.90 |
| CC | 12 (8.4) | 13 (8.8) | 0.7 | | | | 0.3-1.9 | | 0.50 |
| A | 192 (67.1) | 202 (68.7) | 1 (reference) | | | | - | | - |
| C | 94 (32.8) | 92 (31.2) | 0.9 | | | | 0.6–1.2 | | 0.33 |
| ***ESR2* 1082 G>A** | | | | | | | | | |
| **Male** |  |  |  | | | |  | |  |
| GG | 71 (92.2) | 76 (91.6) | 1 (reference) | | | | -- | | – |
| GA+AA | 6 (7.8) | 7 (8.4) | 1.7 | | | | 0.4–6.6 | | 0.44 |
| G | 148 (96.2) | 159 (95.7) | 1 (reference) | | | |  | | - |
| A | 6 (3.8) | 7 (4.3) | 1.7 | | | | 0.5–5.7 | | 0.38 |
| **Female** |  |  |  | | | |  | |  |
| GG | 135 (94.4) | 136 (92.5) | 1 (reference) | | | | - | | – |
| GA+AA | 8 (5.6) | 11 (7.5) | 1.3 | | | | 0.5–3.3 | | 0.63 |
| G | 278 (94.5) | 283 (96.3) | 1 (reference) | | | |  | | - |
| A | 8 (2.7) | 11 (3.7) | 1.1 | | | | 0.5–3.1 | | 0.71 |
| ***PGR* Ins/Del** | | | | | | | | | |
| **Male** |  |  |  | | | |  | |  |
| DD | 60 (77.9) | 77 (79.5) | 1 (reference) | | | | - | | - |
| DI | 16 (20.8) | 6 (7.2) | **0.2** | | | | **0.1–0.7** | | **0.010** |
| II | 1 (1.3) | 0 | - | | | |  | | - |
| D | 136 (88.4) | 160 (96.3) | 1 (reference) | | | |  | | - |
| I | 18 (11.6) | 6 (2.7) | **0.5** | | | | **0.2–1.4** | | **0.020** |
| **Female** |  |  |  | | | |  | |  |
| DD | 121 (84.6) | 131 (89.1) | 1 (reference) | | | |  | | – |
| DI | 21 (14.7) | 16 (10.9) | 0.6 | | | | 0.2–1.2 | | 0.158 |
| II | 1 (0.7) | 0 | - | | | |  | | - |
| D | 263 (91.9) | 278 (94.5) | 1 (reference) | | | |  | | - |
| I | 23 (8.0) | 16 (5.4) | 0.4 | | | | 0.2–1.2 | | 0.256 |
| ***ADRB3*-190 T>C** | | | | | | | | | |
| **Male** |  |  |  | | | |  | |  |
| TT | 61 (79.2) | 66 (79.5) | 1 (reference) | | | | - | | – |
| TC | 16 (20.8) | 16 (19.3) | 0.6 | | | | 0.2–1.8 | | 0.40 |
| CC | 0 | 1 (1.2) | - | | | | - | | - |
| T | 138 (89.6) | 148 (89.1) | 1 (reference) | | | |  | | - |
| C | 16 (10.4) | 18 (10.8) | 1.0 | | | | 0.5–2.0 | | 0.95 |
| **Female** |  |  |  | | | |  | |  |
| TT | 117 (81.8) | 92 (62.6) | 1 (reference) | | | | - | | – |
| TC | 26 (18.2) | 55 (37.4) | **2.8** | | | | **1.5–4.9** | | **0.001** |
| CC | 0 | 0 | - | | | | - | | - |
| T | 260 (90.9) | 239 (81.2) | 1 (reference) | | | | - | | - |
| C | 26 (9.1) | 55 (18.7) | **2.4** | | | | **1.4–3.9** | | **0.001** |
| ***ADRA2A* -1291 C>G** | | | | | | | | | |
| **Male** |  |  |  | | | |  | |  |
| CC | 23 (29.9) | 18 (21.7) | 1 (reference) | | | | - | | – |
| CG | 42 (54.5) | 47 (56.6) | 1.4 | | | | 0.6–3.1 | | 0.33 |
| GG | 12 (15.6) | 18 (21.7) | 2.2 | | | | 0.8-6.0 | | 0.10 |
| C | 88 (57.2) | 83 (50.0) | 1 (reference) | | | | - | | - |
| G | 66 (42.8) | 83 (50.0) | 1.7 | | | | 0.7–4.0 | | 0.197 |
| **Female** |  |  |  | | | |  | |  |
| CC | 38 (26.6) | 35 (23.8) | 1 (reference) | | | | - | | – |
| CG | 75 (52.4) | 70 (47.6) | 0.9 | | | | 0.5–1.6 | | 0.84 |
| GG | 30 (21.0) | 42 (28.6) | 1.4 | | | | 0.7-2.8 | | 0.28 |
| C | 151 (52.7) | 140 (47.6) | 1 (reference) | | | | - | | - |
| G | 135 (47.3) | 154 (52.4) | | 1.5 | | | 0.8–3.0 | | 0.765 |

$ = Gallstone patients; * = Odds Ratio; # = Confidence Interval; Significant values are show in bold.
